# An On-Device Deep Learning Approach to Battery Saving on Industrial Mobile Terminals

**DOI:** 10.3390/s20144044

**Published:** 2020-07-21

**Authors:** Inyeop Choi, Hyogon Kim

**Affiliations:** 1 M3Mobile, Kuikangbyun-Ro 44, Kwangjin-Gu, Seoul 05116, Korea; billychoi@m3mobile.co.kr; 2 Department of Computer Science and Engineering, Korea University, Anam-Dong, Sungbuk-Gu, Seoul 02841, Korea

**Keywords:** industrial mobile terminal, scanner window, frost, deep learning, convolutional neural network (CNN), classification, energy saving

## Abstract

The mobile terminals used in the logistics industry can be exposed to wildly varying environments, which may hinder effective operation. In particular, those used in cold storages can be subject to frosting in the scanner window when they are carried out of the warehouses to a room-temperature space outside. To prevent this, they usually employ a film heater on the scanner window. However, the temperature and humidity conditions of the surrounding environment and the temperature of the terminal itself that cause frosting vary widely. Due to the complicated frost-forming conditions, existing industrial mobile terminals choose to implement rather simple rules that operate the film heater well above the freezing point, which inevitably leads to inefficient energy use. This paper demonstrates that to avoid such waste, on-device artificial intelligence (AI) a.k.a. edge AI can be readily employed to industrial mobile terminals and can improve their energy efficiency. We propose an artificial-intelligence-based approach that utilizes deep learning technology to avoid the energy-wasting defrosting operations. By combining the traditional temperature-sensing logic with a convolutional neural network (CNN) classifier that visually checks for frost, we can more precisely control the defrosting operation. We embed the CNN classifier in the device and demonstrate that the approach significantly reduces the energy consumption. On our test terminal, the net ratio of the energy consumption by the existing system to that of the edge AI for the heating film is almost 14:1. Even with the common current-dissipation accounted for, our edge AI system would increase the operating hours by 86%, or by more than 6 h compared with the system without the edge AI.

## 1. Introduction

The cold storage market size was valued at $29.08 billion in 2016, and it is expected to expand at a compound annual growth rate (CAGR) of 9.9% from 2017 to 2025 [1]. What the trend tells us is that more industrial mobile terminals will be used to scan products in a low-temperature environment such as cold-storage warehouses, and the demand will grow further. These industrial mobile terminals are typically equipped with a film heater in the scanner window. This is to remove the frost that the scanner window may accrue when the terminal comes out of the cold storage to the room temperature outside. The moisture in the outside air condenses on the cold surface of the window when the terminal-carrying worker exits the warehouse. If the scanner window is at a below-freezing temperature, the moisture turns into frost. When the worker returns to the cold warehouse before the frost melts, the terminal cannot function properly because the frost blocks the view of the scanner.

Therefore, the industrial mobile terminals designed to work in low-temperature warehouses typically employ a film heater on the scanner window. When the sensed temperature near the scanner window drops below a certain threshold, the film heater is turned on. However, the temperature and humidity conditions of warehouses are variable. Moreover, the conditions under which the frosting can occur vary widely in terms of the temperature of the device itself and the time duration the device stays in the given environment, etc. Consequently, determining whether the frost has formed based solely on temperature sensors is difficult. Instead of enumerating all frost-forming conditions they need to cope with, existing industrial mobile terminals on the market tend to take a simpler approach that trades energy efficiency for high availability. Namely, they start the film heater slightly above the freezing point and turn it off only when the temperature of the scanner window is even higher. This approach inevitably consumes more energy than necessary, which can shorten the operating time of the device before the device needs to be recharged.

In this paper, we propose an artificial intelligence (AI)-based approach that utilizes deep learning technology to avoid unnecessary scanner heating energy use. At the core is a convolutional neural network (CNN) to ascertain the actual frosting in the scanner window. Wrapping the CNN classifier is the logic that recognizes potential frosting conditions and utilizes the low-cost temperature sensors as in the traditional approach. The wrapper logic for the CNN classifier reduces the probability of incorrect classifications by utilizing the temperature sensors and the image brightness. Although our approach may allow the formation of frost for a very brief amount of time, the melting operation can ready the scanner window before the worker returns to the low-temperature warehouse. In this paper, we implement the CNN on-device and test the proposed approach for its energy-saving effect. The results confirm that our proposed approach reduces the energy consumption almost by half even under a condition that is less favorable to our scheme. We believe that the experience presented in this paper for solving one problem for industrial mobile terminals will be easy to replicate in other problems that they may face in other aspects than energy saving.

The first reason that we adopt deep learning among the many AI techniques is that visually identifying the frost is the most effective and direct solution to the problem. Since AI strives to simulate intelligent human behavior, the CNN classifier for frost images emulates the mobile terminal operator that visually checks for frost to remove it. Another reason to employ CNN is that the industrial mobile terminal uses the scanned image of the 1D or 2D code for its inherent task anyway. It is straightforward to make it available to the CNN classifier to reuse it for the added benefit of detecting frost on the scanner window. Last but not least, using the deep learning approach can be more practical for non-experts in AI such as those in the storage market. Because the approach only requires data for training and because there is strong support in the form of software packages such as Keras [2] and Tensorflow [3], understanding and making machine learning (ML) models for the problem can be avoided. Therefore, instead of using other other machine learning (ML) methods such as fuzzy logic that may solve the problem more indirectly by checking the frost-forming conditions, we take the deep learning approach in this paper. We leave the investigation of more energy-efficient ML approaches to our problem to a future work.

## 2. Related Work

Deep learning models are complex and require a high computational load and memory usage. Therefore, the main platforms to run these models have been powerful computers (e.g., with Graphics Processing Unit (GPU) support) or cloud environments. Recently, however, researchers are realizing the benefits of bringing deep learning towards the edge that includes the devices with less power and resources. Wu et al. [4] claimed that user experience can be improved by bringing machine learning inference to the edge, which reduces latency and makes the device less dependent on network connectivity. Zou et al. [5] observed that in emerging applications such as autonomous systems, human–machine interactions, and the Internet of Things (IoT), it is advantageous to process data near or at the source of the data to improve energy and spectrum efficiency and security, and to decrease latency. Hu et al. [6] explored how computational offloading to the edge improves latency and energy consumption relative to the cloud. Our work in this paper also benefits from running a deep learning model in terms of latency and security without requiring network connectivity. Although our work is not about techniques for optimizing deep learning on mobile devices, we note that many have been developed for that purpose, such as model compression [7], model partition [8], model early-exit [9], edge caching [10], input filtering [11], model selection [12], support for multitenancy [13], and application-specific optimization [14]. For those readers further interested in the architectures, frameworks, and emerging key technologies of deep learning models for training and inference at the network edge, Zhou et al. [15] is an excellent taxonomical reference.

Among deep learning models, CNN is notoriously complex and requires significant computation and memory resources. Because industrial mobile terminals are equipped with limited computation and memory resources and leave little to tasks other than their inherent mission, CNN has been difficult to introduce to mobile terminals. Recently, however, there have been numerous efforts in investigating techniques to make CNN feasible on resource-constrained edge devices [16,17,18]. Efforts for supporting deep learning on mobile platforms have also emerged, particularly for Android and Tensorflow environments [19,20,21]. Thanks to such efforts and progress, the environments for using the CNN inference on our industrial mobile terminal has become mature. In our work, we implement our CNN model in Tensorflow Lite [3] and embed it on our industrial mobile terminal platform. We leverage the state-of-the-art deep learning technology for mobile systems to make the industrial mobile terminals more energy efficient in their intrinsic operations of scanning. As mentioned above, we stress that our work is not about optimizing deep learning models for mobile environments or about reducing the energy consumed by the computation in the deep learning model itself [22,23,24]. Our work focuses on what we can achieve through such optimized deep learning environments. Specifically, we use it to achieve better energy efficiency in removing frost in the scanner window of the industrial mobile terminals used in low-temperature warehouse environments. In the process, we combine deep learning with more traditional logic to maximize the efficacy of the CNN classifier in the operations of our industrial mobile terminal.

## 3. Proposed Method

In this section, we discuss how a typical industrial mobile terminal copes with the scanner frosting problem and contrast it with our proposed approach.

### 3.1. Motivating Example

**Figure 1:**
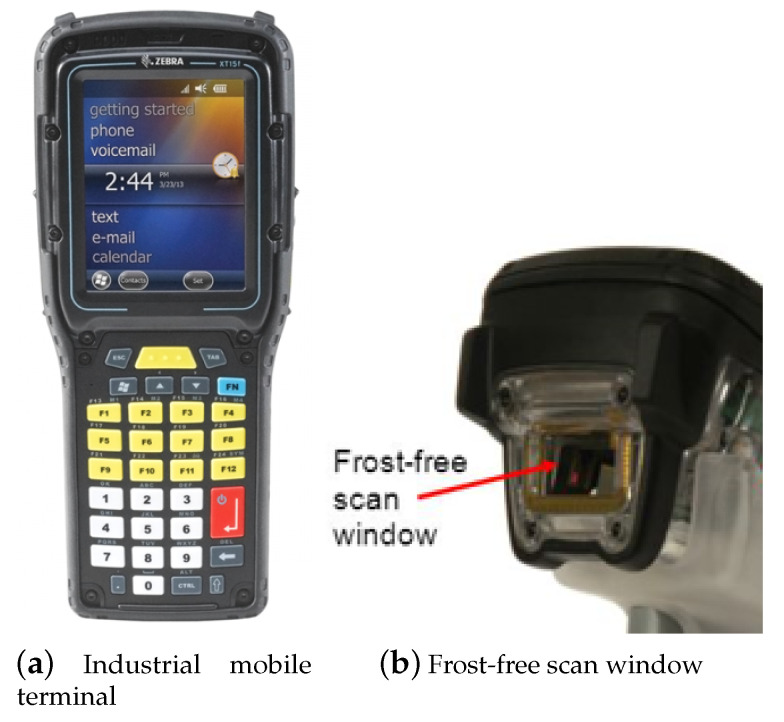
A typical industrial mobile terminal used for low-temperature operation: Zebra Omnii XT15f.

The traditional approach to prevent frost forming is simple—maintain the scanner window well above the freezing temperature. Figure 1 shows a representative industrial mobile terminal, Zebra Omnii XT15f [25], whose scanner window is always kept warm. It has a temperature sensor attached to the scanner window. When the window temperature decreases to 7 ${}^{\circ }$C, it turns on the film heater so that the scanner window temperature does not fall further. Then, when the temperature of the scanner window reaches 34 ${}^{\circ }$C due to the heating, the film heater is turned off. By repeating this pattern whenever necessary, it is possible to prevent frost from forming on the scanner window in any low-temperature operation. The film heater can be in one of two states, on and off, and to the best of our knowledge, no more sophisticated control is employed. Under this simple approach, even if the terminal is moved from a low-temperature warehouse to room temperature, frost will not form. When the terminal enters the low-temperature warehouse, on the other hand, frost forming is not likely a problem as the humidity is typically low. Frost will only form in certain conditions involving the temperature and humidity.

However, this method can incur unnecessary battery consumption. For example, consider a worker using the terminal in a low-temperature warehouse for an extended period of time. Because the scanner window temperature will decrease to 7 ${}^{\circ }$C in due course, the film heater will turn on. Then it will repeatedly turn on and off as the scanner window temperature moves between the two thresholds. But the humidity in the low-temperature warehouse is so low that no frost forms even if the scanner window were left cooled below the freezing temperature. This means that while the worker remains in the warehouse, there will be a significant amount of unnecessary battery drain. We indeed observe with the Zebra Omnii XT15f that, on average, the film heater is turned on for 3 min and turned off for the next 3 min in a typical low-temperature warehouse environment. The pattern repeats throughout the low-temperature operation. This is a problem caused by the inability to precisely identify the frost-forming condition by using only the temperature sensors.

How the energy is used by the film heater can significantly affect the operating time of the industrial mobile terminal. In the illustrative example above, the XT15f terminal [25] has a battery capacity of 5300 mAh. At the system operating voltage of 3.6 V, it can provide up to 19 Wh of energy when the terminal is idle. Its scanner dissipates 400 mA for illuminating the scanned target and 200 mA for decoding the scanned image. At the operating voltage of 3.3 V, and at 300 mA for the average current, the terminal consumes 0.99 W. Adding this to the system power consumption, the total is 1.71 W during the scanning operation. Since the device has 19 Wh in its battery, it will be able to operate the scanner for approximately 11.1 h (Table 1, top). Turning on the film heater on the scanner window further decreases this operation time. It consumes 700 mA at 5 V, or 3.5 W, but assuming that the film heater repeatedly turns on and off for equal amounts of time, the power consumption is 1.75 W. As a result, when the film heater engages, the battery is completely drained in 5.5 h (Table 1, bottom). This could lead to an inconvenience to the user in terms of recharging. This example motivates us to improve the frost detection logic in industrial mobile terminals for low-temperature operation. In other words, by more precisely determining whether there is frost on the scanner window, we can compute the film heater only when frost formation actually takes place.

### 3.2. Proposed Solution

The reason that the traditional approach chooses to use inefficient logic is that it is difficult to account for all of the frost-forming conditions using only the temperature sensors. The condition depends on the temperatures and humidity levels of the scanner window, the device itself, and the room temperature outside the cold warehouse. Given this difficulty, the cheapest and safest approach, but with the worst user experience, would be for the user to visually determine whether the scanner window has the frost and if so, manually operate the film heater until the frost is removed. An energy-inefficient but less cumbersome alternative is the current state of the art, which is turning on the film heater when the scanner window reaches a low threshold temperature. In this section, we propose a third approach that is more energy efficient and yet requires no user intervention.

The proposed idea is to replace the human user in the first approach with artificial intelligence that inspects the scanner window for any frost. Because the scanner is a component that takes a picture of an image of the 1D or 2D code before the image is analyzed by the internal logic, the image is readily available. Utilizing the image, we let an image-classification logic determine whether the image shows frost on the scanner window. In this paper, we implement the image-classification logic with an on-device CNN. To implement the CNN classifier, we proceed in two steps.

1. To make the CNN judge whether or not there is frost in the image from the scanner, we train it with examples (i.e., supervised learning). We take the pictures of actual frost on the scanner window and use them to train the CNN.
2. We incorporate the CNN in the final frost-identification logic that is embedded in the mobile terminal to classify the image data from the scanner in real time.

As to the edge intelligence architecture, there are three options: centralized, hybrid, and decentralized [15]. The intelligence is located in the cloud in the centralized architecture, whereas it is in the edge device in the decentralized one. The hybrid distributes them in the edge and the cloud. For our work, we could run the classification on the cloud, letting the mobile terminal send the image to the cloud and then obtaining the classification from it. However, we use a decentralized architecture in this paper. The reason that the cloud AI approach does not fit the target operating environment for industrial mobile terminals is manifold [26]. To use the cloud AI, we always need to be connected to the network. Moreover, there would be delay by using the remote computing resource over the communication link, when the terminal must work in real time. Using wireless would also consume more power. Last but not least, customers tend to block access to the network in the warehouses from outside, for security reasons. It would require customers to have their own server to locally determine whether the image submitted by the industrial mobile terminal is frosted, which would be an excessive requirement. Therefore, this paper takes the edge AI approach to determine the presence of frost.

To decide when to perform the CNN-based inference, we utilize the temperature sensors on board. We could run CNN periodically to classify the image from the scanner instead, but this can result in inefficient battery use because it will run irrespective of frost forming. Moreover, it would require the scanner to take pictures, which may interfere with the user’s work. Therefore, we choose to perform a temperature sensor-based pre-check for specific conditions that require the CNN-based classification of the scanner image. This will not only help save battery, but also help narrow down the frost-forming conditions and improve decision accuracy.

## 4. Implementation

To demonstrate the usefulness of the solution using the proposed edge AI model, we implement it on our industrial mobile terminal platform. For this, we first train the CNN to learn the frost images on a more powerful computing platform, then embed the trained CNN on the mobile terminal. Furthermore, in order to supplement the CNN classifier that has nonzero classification error probabilities, we implement additional frost-forming condition checking logics that precede the CNN classifier. We package the condition checking logics and the CNN classifier in a single app that runs on the industrial mobile terminal M3Mobile UL20f [27], used as the test platform. Below, we discuss the components of our implementation in more detail.

### 4.1. CNN Classifier for Frost Images

#### 4.1.1. Dataset Preparation

For training, validation, and tests, we use five different sets of images. They are summarized in Table 2.

**Figure 2:**
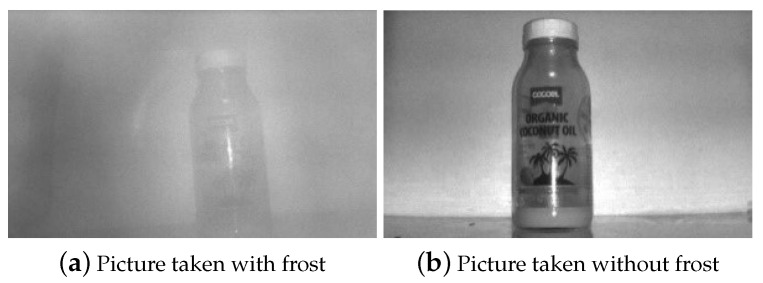
Sample pictures from the dataset.

First, in order to train the CNN, we took 1000 photographs using the test mobile terminal with real frost and 1000 no-frost images. Second, we took two sets of 100 images for validation, each with frost and no frost. Figure 2 shows examples of the pictures in these two datasets.

**Figure 3:**
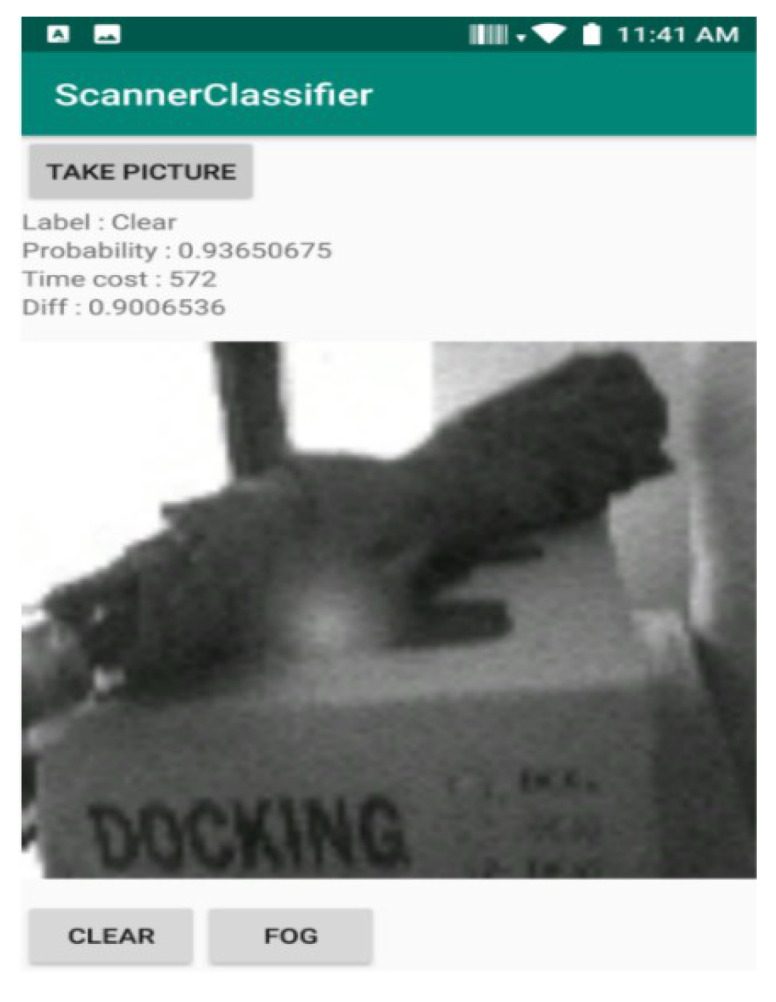
Data collection app: It takes a picture, resizes it, and collects the human-provided label to be associated with it.

The pictures taken with the frosted scanner window were produced after placing the terminal in a cold chamber and then moving it to room temperature. We varied both the temperature of the cold chamber and the time the terminal stays in there when producing the pictures. This was because the extent of frosting differed depending on the temperatures and the cooling time. For the pictures taken without frost on the scanner window, we used various backdrops because the industrial mobile terminal can take the scan against all kinds of backdrops in practice. To facilitate the data collection process, we created a Java application for the terminal, as shown in Figure 3. When the camera in the 2D scanner takes a picture, it appears on the preview screen. Then we provide labels for each figure—with frost on the scanner window, the human user presses FOG, otherwise, CLEAR. Either way, the pictures have a 832 × 640 resolution but are resized by a Java API to 28 × 28 and are saved along with the human-provided label on the terminal. The resolution reduction is in the interest of faster CNN training.

The images in Test I through III are all used in the tests performed on our industrial mobile terminal platform that executes the trained on-device CNN. The images in Test I are used to test the CNN against real frost images under various lighting conditions. Using the result, we identify the lighting conditions under which the CNN can be applied. For other conditions in which the trained CNN has difficulty in the classification operation, such as poor lighting, we use other more simplified classification methods. Test II images are used to confirm the effectiveness of this separation approach and to test the CNN for false negatives. Finally, the images in Test III are no-frost images used to test the CNN for false positives.

#### 4.1.2. CNN Structure Design

**Figure 4:**
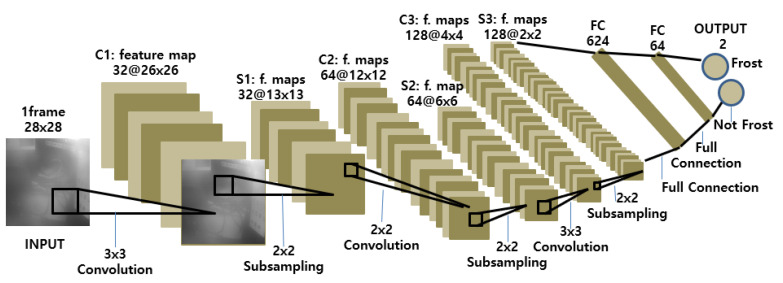
Structure of the convolutional neural network (CNN) used for frost detection.

We designed the structure of our CNN classifier as in Figure 4. It takes a 28 × 28 image input from the scanner window and passes it through three convolutional hidden layers (C1, C2, C3) and two fully connected (FC) layers. The convolutional layers have 32 3 × 3, 64 2 × 2, and 128 3 × 3 filters, respectively. No padding is used, and the stride is set to 1. The pooling layers all use 2 × 2 max pooling. The first fully connected layer has 625 neurons and the second, 64 neurons. The output layer has two neurons that predict “frost” and “no frost,” respectively. We could achieve higher classification performance with a higher image resolution that would require a deeper, larger network, but to reduce the complexity of the network for the limited industrial mobile terminal platform, we settled for the shown CNN structure.

The model complexity of the CNN model can be represented by the number of parameters in each layer. Table 3 enumerates the complexity of each layer in the model. In total, our CNN model has 442,687 parameters.

#### 4.1.3. Training and Validation

We trained the CNN offline on a laptop computer that has more computing power than industrial mobile terminals. For this training step, we used the open neural network library Keras [2], with Tensorflow [28] as the back end. We used the Adam optimizer [29] and Hyperband [30] to optimize the hyperparameters through multiple test runs. Consequently, we found that the best configuration is as follows:

- Batch size = 30
- Epochs = 20
- Dropout = 0.1

**Figure 5:**
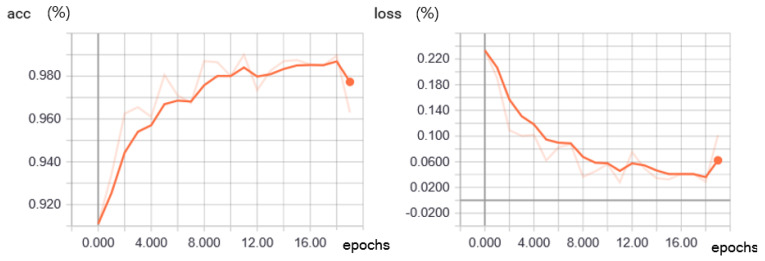
Training accuracy (**left**) and training loss (**right**) of the frost classifier.

Figure 5 shows the accuracy and the loss of the training for the frost classifier. We measured the accuracy of the trained CNN by using the 2000 images mentioned in Section 4.1.1 with the model.evaluated() function in Keras. At epoch 18, it reaches 98.95%, when the loss is 0.029%. Applying the trained model to the 200 validation images, we obtained 190 correct decisions out of the 200, or 95% accuracy. For the loss function, we used categorical cross-entropy, available in Tensorflow. After the CNN was transplanted to the industrial mobile terminal platform, more extensive tests were performed against a set of test images different from the total of 2200 images used in the training phase, the results of which appear in Section 4.4.

### 4.2. Transplanting the Trained CNN to an Industrial Mobile Terminal

**Figure 6:**
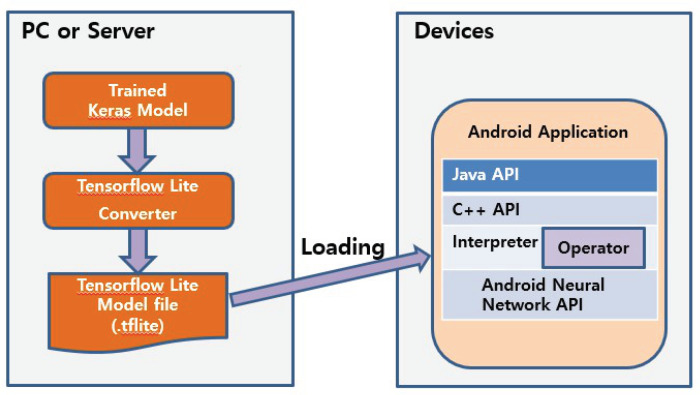
Loading the trained model to our industrial mobile terminal.

In order to load the trained CNN model to the resource-limited industrial mobile terminal platform, we transformed the trained model by using TensorFlow Lite. TensorFlow Lite changes the learned Tensorflow model to a TensorFlow Lite file format. The file, converted_model_keras_fog.tflite, is only 395 KB and can be readily accommodated by our test terminal. Figure 6 shows the loading process.

**Figure 7:**
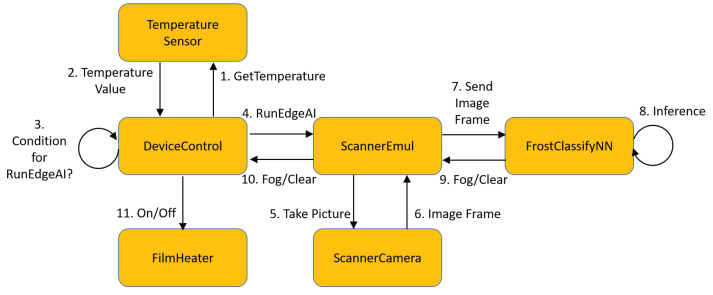
System state diagram.

As a wrapper for the CNN classifier, we programmed an Android application as the OS used in the test terminal is Android. The Android application performs two services. The first is DeviceControl, which reads the sensors and controls the film heater. It reads the temperatures once every second to engage the edge AI if necessary and turns on the film heater when the edge AI reports frost. In addition, it turns off the film heater if the scanner window temperature is over a threshold. The second service is ScanEmul, which runs when the DeviceControl service determines through the temperature sensors that the edge AI needs to engage. For this, ScanEmul loads converted_model_keras_fog.tflite and then serializes it into the FlatBuffers format before operation. Finally, it executes the model through the TFLite interpreter. The ScanEmul service takes a picture by using the camera in the scanner module. This original image has a 832 × 640 resolution, but the service resizes it to a lower resolution (28 × 28) so that the CNN can easily process it on-device. It then engages the CNN on the resized image and returns the classification result to the DeviceControl service. The state machine that depicts these interactions appears in Figure 7. A typical chain of transitions is: DeviceControl/GetTemperature → TemperatureSensor/Temperature Value → DeviceControl/Condition for RunEdgeAI? → DeviceControl/RunEdgeAI → ScannerEmul/Take Picture → ScannerCamera/Image Frame → ScannerEmul/Send Image Frame → FrostClassifyNN/Inference → FrostClassifyNN/Fog → ScannerEmul/Fog → DeviceControl/On. The TFLite interpreter operates on the Android Neural Network Runtime through the Android Neural Network API [31]. Both the interpreter and the Android Neural Network run as Android application processes. The Android Neural Network is supported from Android 8.1.

### 4.3. Temperature Condition Checking Logic

The second piece of the proposed system is the frost-forming condition check using the temperature sensors. If this check says negative, the CNN classifier is not invoked. The precondition check using the temperature sensors incurs a much lower energy cost than the CNN classification, and we can save energy by turning to the CNN only when we need “visual” confirmation by the edge AI. Moreover, it prevents the CNN from continuously taking pictures through the scanner, which would interfere with the normal scanning operation performed by the human worker.

**Figure 8:**
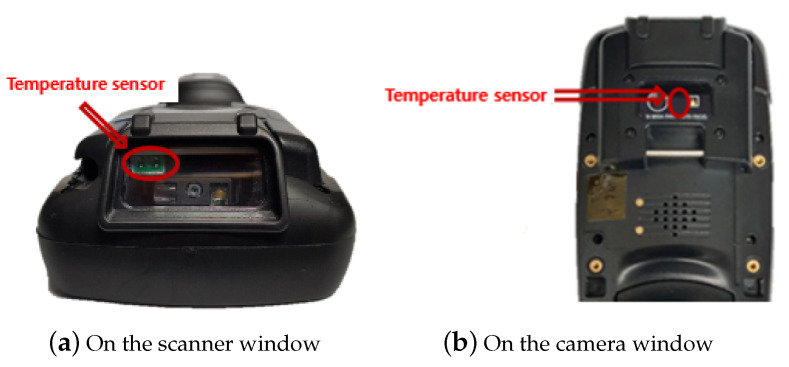
Temperature sensors on the test platform (UL20f).

Our test terminal has two temperature sensors. One is mounted just beneath the scanner window to measure the temperature of the scanner window itself (Figure 8a). The other temperature sensor is attached to the camera (different from the scanner) window at the top of the terminal to measure the ambient temperature (Figure 8b). The temperature sensors send the measured temperature values to the Android system once every second. The film heater on the scanner window generates heat and removes the frost on the scanner window when the current flows through it. The film heater is controlled by the connection to the general-purpose input/output (GPIO) pin of the application processor (AP).

The necessary condition to make the scanner window frost is that the industrial mobile terminal’s internal temperature remains below the freezing point when the device is carried to a much warmer ambient temperature. To detect this condition that calls for the engagement of the CNN, we require that

1. Both sensors at the scanner window and at the camera window report below-freezing temperatures;
2. At least one of the temperature sensors records the temperature rising by more than a threshold from the measurement in the last second. The threshold is set to 1 ${}^{\circ }$C in our test system.

**Figure 9:**
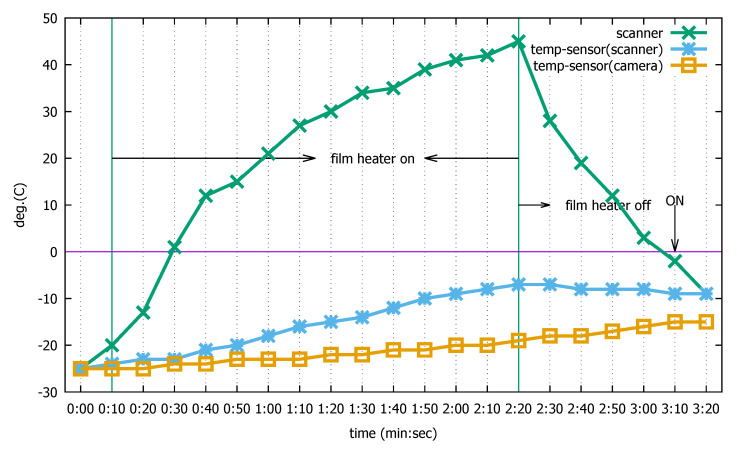
Temperature changes on two sensors when the industrial mobile terminal turns on and then turns off the film heater; without edge artificial intelligence (AI) logic.

The reason that we use this logic can be explained through an experiment illustrated in Figure 9, which shows the temperature changes on the scanner surface (“scanner”) and at the two sensors when our approach is employed. In this particular test, we measure the surface temperature using a separate infrared thermometer because there is no on-device temperature sensor for it. Meanwhile, the scanner temperature sensor measures the temperature beneath the scanner (“temp-sensor(scanner)”) and the camera temperature sensor measures the temperature beneath the camera (“temp-sensor(camera)”).

In the graph, at t=0:00, the device is taken out of a cold chamber at −25 ${}^{\circ }$C and placed at room temperature. The frost starts to form since the device temperature is below freezing, as testified by the three different temperature measurement values. At t=0:10, the scanner sensor temperature rises by 1 ${}^{\circ }$C, which triggers CNN classification according to the two required conditions above. As the CNN returns the positive identification of frost formation, the film heater is turned on. Once the heater is turned on, it is kept on for a sufficient duration to melt the frost. In our test system, this is 130 s. During the heating, the surface temperature rises to as high as 45 ${}^{\circ }$C, enough to melt the frost and evaporate the remaining moisture. However, the two temperature sensors on the terminal do not rise as quickly because they are located inside the device and the device is still cold. In particular, when the film heater turns off (t=2:20) after the 130-second heating interval, the two sensors still sense negative temperatures. Moreover, in the absence of heating, the scanner window sensor sees the temperature begin to drop due to the temperature difference with the cold device body. However, the camera temperature sensor keeps steadily rising due to the higher room temperature, irrespective of the operation of the film heater located relatively far away. When it rises by 1 ${}^{\circ }$C, it again satisfies the two conditions described above, and the CNN is consulted. However, this time, the CNN returns a negative because the frost has been removed by the 130-second heating. Then, CNN consultation happens every 1 ${}^{\circ }$C rise at the camera temperature sensor as long as the two conditions are met. At t=3:10, the CNN finally reports a positive, as the surface temperature of the scanner window is now below freezing and frost begins to form again. As we can see in this illustration, both temperature sensors are essential to steer the edge AI logic.

Although the small temperature difference threshold of 1 ${}^{\circ }$C in the second requirement can lead to false positives, a larger threshold can delay the engagement of the CNN classifier, so we take the calculated risk that will be further suppressed by an additional logic to be discussed in Section 4.4.1. Should this happen, it may lead to some unnecessary power consumption, but it is our design philosophy that a false positive is better than a false negative that will more directly affect the user experience in the form of frosting.

### 4.4. Image Brightness Checking Logic

The false negative being the biggest user experience (UX) issue, we set the CNN decision threshold so that the false negatives are minimized at the cost of increased false positives. Consequently, the proposed system scarcely fails to identify frost. Then, the false positives are also mostly filtered by the frost-forming condition check discussed above, and the final energy cost due to the false positives is small. This aspect will be discussed in Section 4.4.2.

#### 4.4.1. Coping with False Negatives

**Figure 10:**
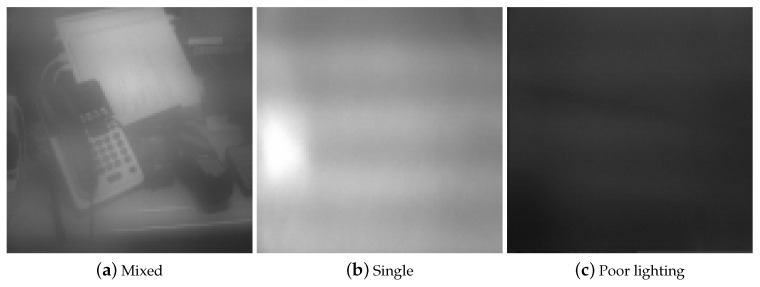
Three types of images.

In order to evaluate the false negative rate of the proposed system, we tested our system installed on the mobile terminal under the frost condition. The test was conducted against three types of images, as shown in Figure 10.

**Figure 11:**
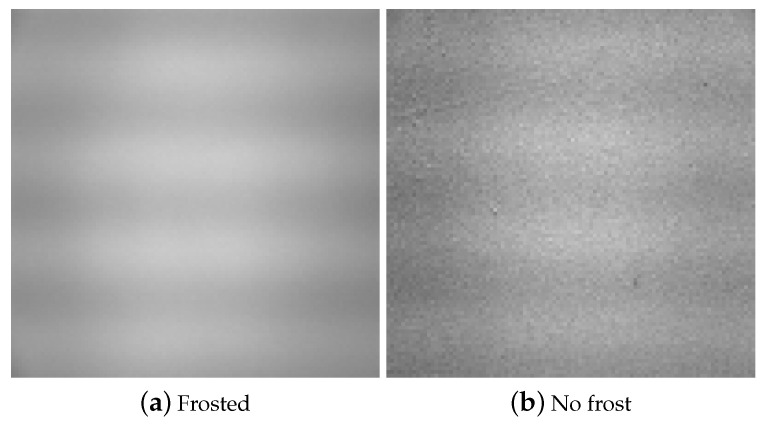
Frost and white/gray no-frost images are hard to distinguish.

“Mixed” refers to the case where various objects with different colors are in the image. “Single” has no noticeable objects in the image, for example, a picture of a wall. With “poor lighting”, the image is simply black. The single-color case can be further divided into two subcases: white/gray and other colors. The former poses the greatest difficulty due to the similarity to frost, as Figure 11 illustrates. As for the latter, they have less brightness and are less difficult for the CNN to make the decision.

There are two cases that can potentially increase the false-negative rate. The first is when the CNN classifies white or gray images. In this case, our experiment with these images in the training phase produced a false-negative rate of over 50%. We labeled them “frost” so that high false negatives are prevented. As a consequence, the CNN is trained to classify white or gray no-frost images as “frost”, causing a problem of potentially producing a high number of false positives. However, most of the false positive problems can be prevented by the aforementioned temperature-checking logic. Only those white or gray no-frost images taken under the two required conditions specified in the previous section will be falsely classified as “frost”. In essence, we transform the false-negative problem for white/gray images to a false-positive problem that we address with the temperature-checking logic. Section 4.4.2 below indeed shows that the resulting actual false-positive rate is very low.

The second group of false-negative cases arises when the CNN classifies the frost under poor lighting. In this case, the image is nearly black, as in Figure 10c, or dark and barely visible for mixed-color objects. For instance, we tested 30 frost images with single or mixed colors under poor lighting, and all were classified as “no frost”. For these cases, we cannot rely on the CNN, so using it leads to incorrect classifications and energy waste for the complex computation by the CNN. Therefore, we need to set aside these classes of images, for which we use a classification method other than the CNN. Specifically, we use an image brightness analysis for these. For this, we first convert the 28 × 28 image to grayscale. Then we obtain the maximum and minimum brightness across all 784 pixels, the gap between which is the brightness difference $\delta_{B}$. We also compute the average brightness $\bar{B}$ of the pixels. Likely frost images will have a very small $\delta_{B}$ and high brightness $\bar{B}$ under good lighting. This is because any objects in the image are blurred by the frost. The reason for the large $\bar{B}$ is that the color of frost produces white pixels in the entire image. To calibrate this logic, we tested our trained model against 551 frosted images taken by the test mobile terminal (i.e., Test I image set), for which we obtained 69 false negatives. Analyzing these false negatives, their $\bar{B}$ and $\delta_{B}$ are distributed as in Table 4. The poor lighting cases dominate the false negatives, as expected. In this case, the false negatives can be addressed by small $\bar{B}$ and $\delta_{B}$ values. There are not many false negatives in well-lit images because they exclude the white/gray images that the CNN is trained to classify as “frost”, as we discussed above.

Based on this experimental result, we position the following logic before the CNN classification. The poor lighting images with $\bar{B}<0.2$ and $\delta_{B}<0.3$ are classified as “frost” to avoid potential false negatives. For these cases, the CNN classification is skipped. The approach will inevitably produce false positives, but we attempt to reduce the possibility by applying an additional logic to be discussed in Section 4.4.2. For all other brightness conditions not covered by the two cases, we let the CNN decide as before.

After incorporating the temperature check and the image brightness check before the CNN classifier, we test with another 600 frost images (i.e., Test II dataset), which produces the classification result as shown in Table 5. Because typical warehouse environments are relatively well lit, we set the ratio of good lighting to poor lighting images in the dataset to 9:1 in this test. The ratio of mixed to single colors was set to 5:5.

Note that the white/gray images under good lighting and the poor lighting cases are forced into the “frost” category through the CNN training and through Algorithm 1, respectively. We can see that only 9 out of 270 (3.3%) mixed-color images under good lighting have been incorrectly classified, whereas all other cases are correctly classified. In total, 591 out of 600 test images were correctly classified, with a 1.5% error rate. Note that some of these numbers are blind decisions based on the image brightness check. The image brightness checking logic helps to decimate the false negatives but in return, it can increase the false positives. We discuss this aspect below.
**Algorithm 1** Suppressing false negatives through image brightness examination 1: **if**
$\bar{B}<0.2\wedge \delta_{B}<0.3$
**then**▹ Poor lighting: just say “frost” to avoid false negative 2:     decision = “frost” 3: **else**▹ Favorable condition to run CNN classification 4:     decision = CNN_classification 5: **end if**

#### 4.4.2. Coping with False Positives

A false positive is when the CNN classification is “frost” where the ground truth is not. It is not as critical as a false negative, but it causes unnecessary battery use. Above, we forcefully classified both the white/gray color images and all images under poor lighting as “frost” images, risking false positives to prevent the more serious false negatives. Applying the changes specified in Algorithm 1, the false-positive test results against no-frost images under good lighting (i.e., Test III dataset) are shown in Table 6. The accuracy is as high as 98.5% and 99.3% for mixed- and single-color images, respectively. The small fraction of incorrect decisions by the CNN can be further prevented by the temperature-checking logic (Section 4.3) that precedes the CNN classifier. The poor lighting and white/gray images that have no frost are not tested here because they will also be mostly filtered by the temperature-checking logic. Finally, the remaining false-positive possibility only leads to additional energy costs that should be much smaller than in the traditional approach.

### 4.5. Integrated Logic

**Figure 12:**
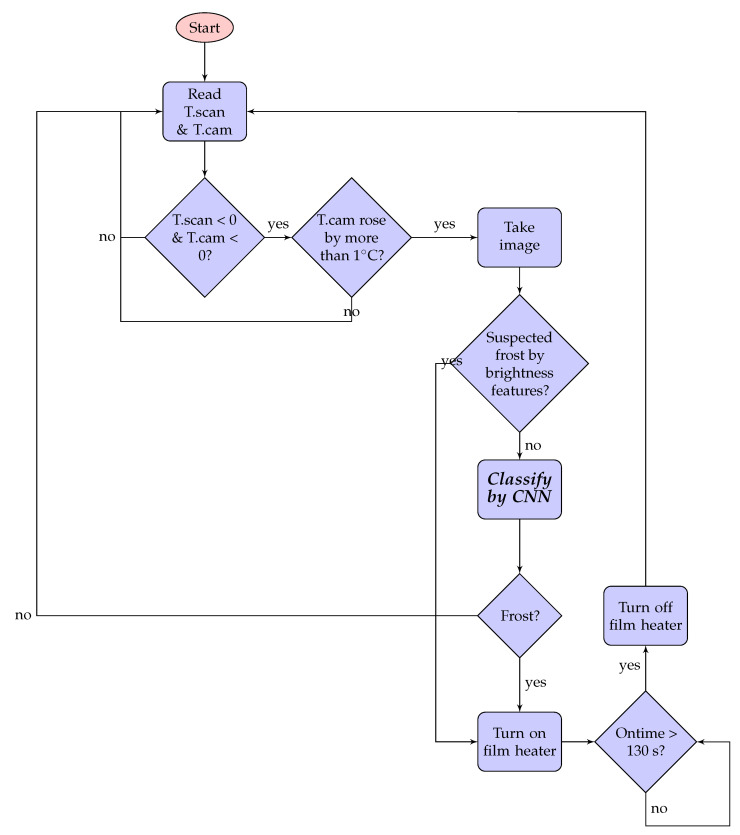
Proposed edge AI algorithm.

Figure 12 summarizes the proposed algorithm that integrates the temperature-checking logic, the image brightness checking logic, and the CNN classifier. Note that the CNN is preceded by two checking logics, so that it is invoked only when necessary. The film heater works for 130 s once it turns on, as we **found** through repeated tests on our platform that this prevents frost-forming, except for the first 10 s when the mobile terminal is moved from the cold warehouse to room temperature.

## 5. Energy-Saving Performance of the Proposed System

**Figure 13:**
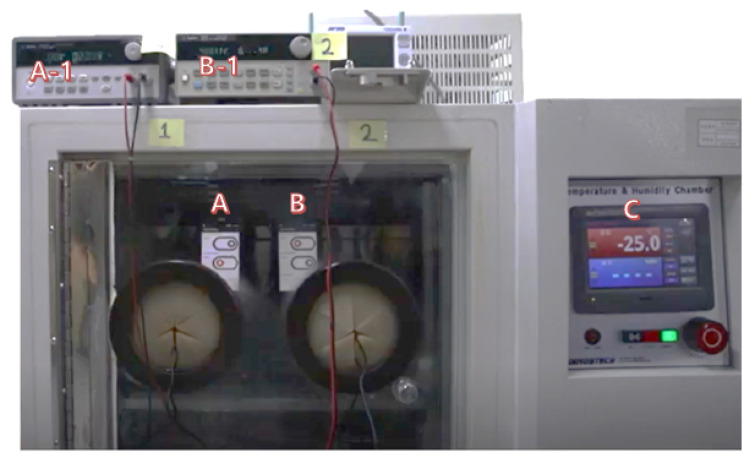
Cold-chamber experiment (A: Terminal with existing method; B: Terminal with edge AI method; A-1 and B-1: DC power supplies for measuring the current at Terminal A and B, respectively; C: Cold-chamber controller maintaining −25 ${}^{\circ }$C).

In order to measure the energy-saving impact of the proposed algorithm, we tested the prototype in a realistic setting. The setting emulates a typical working environment of the industrial mobile terminal user, who spends 50 min in a cold warehouse after she takes a 10-minute break in room temperature. In reality, the user will keep this working schedule during an entire work day (e.g., 8 h), but we emulate only a single (1-hour) cycle in our experiment. We assume that a cold warehouse is maintained at −25 ${}^{\circ }$C, and the outside room temperature is 22 ${}^{\circ }$C. In order to emulate the cold warehouse in the lab test, we used a cold chamber as shown in Figure 13. Since we cannot freely operate the terminal in the cold chamber to emulate the scanning operation, the energy consumption measured in this cold chamber experiment does not account for the scanner operation. We therefore separately account for the scanner energy consumption in the measured values later.

We placed two industrial mobile terminals in the cold chamber for 50 min. One was the traditional terminal that always turns on the film heater when the temperature measured at the scanning window decreases to 7 ${}^{\circ }$C. The other terminal was equipped with our proposed edge AI logic. Because we needed to modify the internal logic of the industrial mobile terminal to install our proposed system, we employed one such system, UL20f from M3Mobile [27]. This industrial mobile terminal is equipped with a film heater that consumes 5 V × 400 mA. For the traditional approach, we set the industrial mobile terminal to turn on the film heater when the temperature of the scanner window decreased to 7 ${}^{\circ }$C. The industrial mobile terminal with the edge AI executed the algorithm in Figure 12. After cooling the industrial mobile terminals in the cold chamber for 50 min, we took them out and left them at room temperature. While the industrial mobile terminals were in and out of the cold chamber, we measured their current dissipation by using Agilent E3640A DC Power Supply (see Figure 13). The measured energy consumption of the industrial mobile terminals includes that used to drive the system and the LCD monitor. But this condition equally applies to the two compared systems, so the difference between their energy consumption, not the absolute values, should be noted.

**Figure 14:**
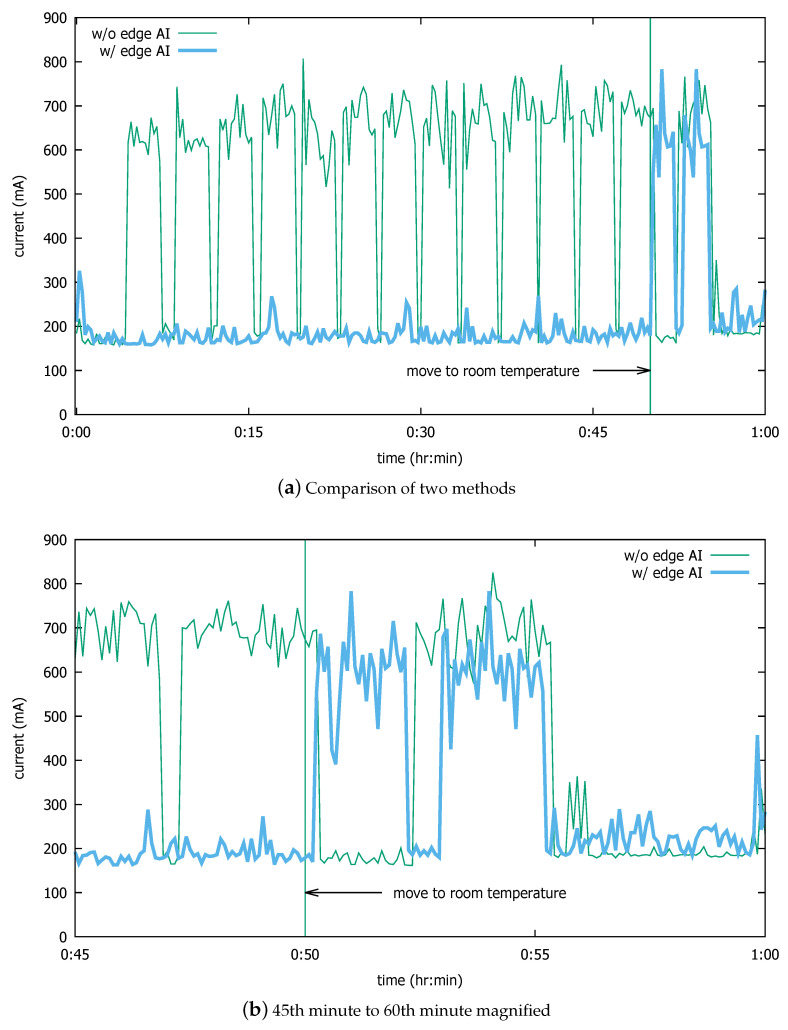
Comparison of current dissipation with and without the edge AI.

Figure 14a compares the currents dissipated by the two approaches. At time t=0, the terminals are placed in the cold chamber. At around t=4 min, the traditional terminal (thin curve) turns the film heater on first. Then it goes through repeated current surges due to the heating operations. The lower power on the tested device does not push the temperature as high as XT15f (i.e., 34 ${}^{\circ }$C), so the heating operation is instead aborted by the 3-minute timer; but the operation soon resumes as the temperature reaches 7 ${}^{\circ }$C again.

In contrast, the edge AI terminal (thick curve) successfully suppresses the unnecessary heating while in the cold chamber, even though the temperature sensors report low temperatures. The algorithm in Figure 12 effectively prevents unnecessary heat-film operation. Instead, it turns on the film heater twice, at around t=50 and t=53 min. This is after the terminal is taken outside of the cold chamber, when the temperature sensors report a temperature increase of 1 ${}^{\circ }$C. After approximately 10 s in room temperature, the device turns on the film heater. Figure 14b shows in more detail the current dissipation of the compared methods after the industrial mobile terminals are taken out of the cold chamber. The reason the proposed method turns on the film heater twice after the terminal returns to room temperature is that the larger part of the device is still cold and makes the heated part (i.e., scanner window) cool down after the heater is off, as shown in Figure 9. This makes the proposed system trigger another heating session because the frost forms and the CNN positively identifies it. However, our method saves much more energy during the low-temperature operation. The traditional system also turns on the heater after it enters the room as the device cools down the heat from the last heating session that happened in the cold chamber.

Table 7 shows the measured numbers related to the energy consumption aspects of the compared systems. Note that so far, we have assumed the idle state where only the system and the LCD incur current consumption, except for the film heater. In reality, however, the 2D scanner is activated for each scan operation. The 2D scanner incurs 300 mA at 3.3 V on the test device. Since we cannot know prior how frequently the scanner will be activated in reality, we conservatively assume that it will be constantly used. Note that under this assumption, the energy consumption ratio of the existing method to that of the edge AI will be underestimated. Despite this biased assumption, however, Table 7 shows that a total of 525 mAh was consumed with the existing model and 214 mAh with the edge AI, a 2.5-fold reduction. These numbers include the common current dissipation for the system and the liquid crystal display (LCD), as well as the 2D scanner, but the net ratio of the energy consumption by the existing system to that of the edge AI for the heating film is almost 14:1. Even with the common current dissipation accounted for, the edge AI system increases the operating hours by 86%, or by more than 6 h.

## 6. Discussion

In the broader context, this paper shows where the edge AI is best applied in edge devices. As artificial intelligence is designed to substitute human intelligence, if there is a component that is performed by human users due to the difficulty of automatization, that is where the adoption of edge AI should be seriously considered. In this paper, this is exemplified by the replacement of the visual recognition of frost on mobile terminals with image-classification AI. As for the implementation of such an edge AI component, the paper also shows that, thanks to the upgraded specification in the mobile terminals and the rapid technological advances in AI technology, this has become more than feasible. Finally, the paper also shows that it pays off to focus edge AI on the most costly aspect. For instance, this paper takes on the film heater that can consume up to half of the battery in cold warehouse operation, regardless of the utilization of the scanner. As a result of applying edge AI on this aspect, the paper shows that the operation time can be extended by almost 86%.

## 7. Conclusions

Today’s industrial mobile terminals have enough computing capacity to accommodate AI to aid their specific tasks. Thanks to the progress of AI technology, deep neural networks for such specific tasks can be developed in small sizes to fit the memory and computing capacity of industrial mobile terminals. In this paper, we demonstrated an application of edge AI technology to develop an intelligent energy-saving strategy for devices used in low-temperature environments such as warehouses. The scanner window of the terminals can be frosted by differences in the temperature and humidity of the operating environments when the user moves in and out of warehouses. The difficulty in developing precise defrosting logic arises from the variety of temperature–humidity combinations that the industrial mobile terminal may work in. Thus, the conventional approach simply heats up the window whenever the scanner window temperature approaches the freezing temperature. However, the availability of image-classification technology empowered by a light-weight deep neural network allows us to determine more precisely when to turn on the film heater. We demonstrated that this enables us to save battery power, and for a typical industrial mobile terminal, this can extend the operating time almost by a factor of two. In the future, we believe that edge AI will help improve other aspects of field devices such as the industrial mobile terminals that we considered in this paper.

## Figures and Tables

**Table 1:** Power consumption of the existing system: with film heater off (top) and on (bottom).

	Avg. Current	Voltage	Avg. Power Consumption	Battery Life
System	200 mA	3.6 V	0.72 W	11.1 h (=19 Wh/1.71 W)
2D scanner	300 mA	3.3 V	0.99 W	
System	200 mA	3.6 V	0.72 W	5.5 h (=19 Wh/3.46 W)
2D scanner	300 mA	3.3 V	0.99W	
Film heater	700 mA	5.0 V	1.75 W (at 50%)	

**Table 2:** Five sets of images used in this paper.

Dataset	No. of Images	Test Device	Description
Training	2000	PC	1000 frost images + 1000 no-frost images
Validation	200	PC	100 frost images + 100 no-frost images
Test I	551	Mobile terminal	Frost images under various lighting conditions
Test II	600	Mobile terminal	Frost images under various lighting conditions
Test III	405	Mobile terminal	No-frost images under various lighting conditions
Total	3756		

**Table 3:** Model complexity.

Layer	Tensor Size	Weights	Biases	Number of Parameters
Input image	28 × 28 × 1	0	0	0
Convolution-1	26 × 26 × 32	288	32	320
Subsampling-1	13 × 13 × 32	0	0	0
Convolution-2	12 × 12 × 64	8192	64	8256
Subsampling-2	6 × 6 × 64	0	0	0
Convolution-3	4 × 4 × 128	73,728	128	73,856
Subsampling-3	2 × 2 × 128	0	0	0
FullyConnected-1	624 × 1	319,488	624	320,112
FullyConnected-2	64 × 1	39,949	64	40,013
Output	2 × 1	128	2	130
Total		441,773	914	442,687

**Table 4:** Image brightness characteristics in false-negative cases.

	$\bar{B}$	$\delta_{B}$	Cases
Good lighting	>0.4	>0.5	0
		0.4–0.5	3
		<0.4	5
Poor lighting	<0.2	<0.3	6
	0.1–0.2	<0.2	15
	<0.1	<0.1	40
Total			69

**Table 5:** False negative test results under the frost condition with the trained CNN and image brightness check.

	Mixed Colors		Single Color		
	Good Lighting	Poor Lighting	Good Lighting		Poor Lighting
			White/Gray	Other Colors	
No. of tests	270	30	135	135	30
Correct (“frost”)	261	30	135	135	30
Incorrect (“no frost”)	9	0	0	0	0
Accuracy (%)	96.7	100	100	100	100

**Table 6:** False-positive test results under the no-frost condition and under good lighting with the trained CNN on the test terminal.

	Mixed Colors	Single Color
No. of tests	270	135
Correct (“no frost”)	266	134
Incorrect (“frost”)	4	1
Accuracy (%)	98.5	99.3

**Table 7:** Energy-saving effect of the edge AI in scanning operation.

	Avg. Current	Voltage	Power Consumption	Battery Life
System (Idle)	190 mA	3.6 V	0.68 W	-
2D Scanner	300 mA	3.3 V	0.99 W	
Film heater (traditional)	335 mA	5 V	1.68 W	7.2 h (=24 Wh/2.36 W)
Film heater (edge AI)	24 mA	5 V	0.12 W	13.4 h (=24 Wh/0.8 W)
